# Maternal-Fetal Characteristics of Pregnant Women With Severe COVID Disease and Maternal-Neonatal Characteristics of Neonates With Early-Onset SARS-CoV-2 Infection: A Prospective Data Analysis

**DOI:** 10.7759/cureus.27995

**Published:** 2022-08-14

**Authors:** Kavita Khoiwal, Anoosha K Ravi, Anmol Mittal, Bhrajishna Pallapothu, Mayank Priyadarshi, Amrita Gaurav, Rajlaxmi Mundhra, Latika Chawla, Anupama Bahadur, Jaya Chaturvedi

**Affiliations:** 1 Obstetrics and Gynaecology, All India Institute of Medical Sciences, Rishikesh, Rishikesh, IND; 2 Neonatology, All India Institute of Medical Sciences, Rishikesh, Rishikesh, IND

**Keywords:** sars-cov-2, mother-to-child transmission, maternal mortality, intrauterine fetal demise, severe covid-19

## Abstract

Introduction

COVID-19 and its mutants have significantly impacted the health care system, claiming numerous lives and adding to the morbidity. The data are scarce to describe the effect of disease severity on pregnancy outcomes, the possibility of mother-to-child transmission, and neonatal outcomes of COVID-positive babies. This study aimed to report the maternal and fetal characteristics of pregnant women with severe COVID disease as well as maternal and neonatal characteristics of neonates with early-onset SARS-CoV-2 infection.

Materials and methods

This is a prospective data analysis of pregnant women with severe COVID disease and neonates with early-onset SARS-CoV-2 infection. The disease parameters including demographic data, clinical presentation, investigations, management, and maternal and neonatal outcomes were recorded and analyzed.

Results

India has faced three waves till now. At the study center, a total of 165 (60, 68, and 37 in the first, second, and third waves, respectively) COVID-positive pregnant women were admitted during all three waves. No severe COVID disease with pregnancy was noted in the first and third waves.

During the second wave (March to June 2021), 15 pregnant women were found to have severe COVID disease. All of them had COVID-related symptoms, with the majority requiring supplementary oxygen at presentation. Nine of these women had intrauterine fetal demise at admission. Nearly 73% were in their second trimester, and the rest were in the third trimester. There was raised total leukocyte count and alanine transaminase in 73% and raised aspartate transaminase in all cases. All of them were admitted to the intensive care unit. Two women in their third trimester had a termination of pregnancy by cesarean section, and one of the neonates had early neonatal death due to perinatal asphyxia. Both the neonates were COVID-19 positive. Eleven women with critical illness succumbed to the disease.

No neonate was found to have early-onset SAR-CoV-2 infection during the first and third waves. Only 11 neonates tested positive for SARS-CoV-2 at the time of birth during the second wave. None of them had any COVID-related symptoms. Preterm birth was reported in four cases. The average Apgar scores at 1 and 5 minutes were 6.9 and 8.09, respectively. The average birth weight was 2,551.81 grams. All neonates were initially kept in the neonatal intensive care unit. Out of 11, four neonates required treatment in the form of positive-pressure ventilation, chest compressions, high-flow nasal oxygen, and non-invasive and invasive ventilation. Neonatal mortality was documented in two cases. Six mothers had one or more positive results in either amniotic fluid, placental membrane, or vaginal or cervical swab, highlighting the possibility of antepartum or intrapartum transmission.

Conclusion

Severe COVID disease during pregnancy was associated with high rates of intrauterine fetal demise and maternal mortality. Raised liver enzymes might be taken as a predicting factor for severe disease. On the other hand, early-onset neonatal SARS-CoV-2 infection is mostly asymptomatic and has a good prognosis. Additionally, mother-to-child transmission of SARS-CoV-2 is possible in the antepartum and intrapartum periods.

## Introduction

COVID-19 pandemic with its multiple waves of infection is still nowhere close to an entity of the past. Another wave with a mutant variant is constantly lurking around and threatening the health care system. India has already witnessed three waves of this infection, and the disease has claimed numerous lives and not just added to the morbidity. Still, the guidelines on its diagnosis or management are not clear. Like always, vulnerable groups such as pregnant women lag behind the rest of the general population in vaccination efforts and management guidelines. Because of physiological alterations in immune and cardiopulmonary systems, pregnant women have been more susceptible to viral respiratory infections [1,2] like SARS-CoV-2. Among the three waves in India, the second had a much more significant burden on all forts. There was a peak of 4 lakh cases per day during this wave. There have been studies comparing the pregnancy cohort with the non-pregnancy cohort for susceptibility and severity of this infection, one of which was conducted at the same study institute wherein it was mostly observed that pregnancy as such though more susceptible does not add on to the risk of severity or outcome of the disease [3]. As observed in other countries [4,5], the second wave in India witnessed a much alarming rate of infection in pregnancy and associated mortality.

The occurrence of neonatal SARS-CoV-2 infection is rare and less studied [6,7]. The data are scarce to describe the effect of early-onset SARS-CoV-2 infection on neonatal outcomes and the possibility of mother-to-child transmission.

This study aimed to report the maternal and fetal characteristics of pregnant women with severe COVID disease as well as maternal and neonatal characteristics of neonates with early-onset SARS-CoV-2 infection.

## Materials and methods

This was a prospective cohort study conducted at a tertiary referral center in North India. All pregnant women with severe or critical COVID disease and neonates with early-onset SARS-CoV-2 infection were included in the study.

Per the institutional policy, COVID-19 testing was done in all pregnant women at admission by reverse transcriptase-polymerase chain reaction (RT-PCR) in an Indian Council of Medical Research (ICMR)-approved laboratory. All pregnant women with COVID-19 infection were screened for severe or critical disease as defined by the Chinese Center for Disease Control and Prevention [8]. Severe infection was defined as dyspnea, respiratory rate > 30 per minute, blood oxygen saturation ≤ 93% on room air, and partial pressure of arterial oxygen to fraction of inspired oxygen 50% within 24 to 48 hours on chest imaging. The critical disease was defined by respiratory failure, septic shock, and/or multiple organ dysfunction or failure. Prior permission for data collection and analysis was taken from the Institutional Ethical Board (AIIMS/IEC/20/559).

Study personnel collected data including demography, symptomatology, known co-morbidities, period of gestation, pregnancy-associated complications, details of investigations and management, duration of hospital stay, intensive care unit (ICU) stay, ventilatory requirement, and pregnancy outcomes including maternal and neonatal outcome from the time of admission to discharge or death while admitted. Vaccination was not recommended for antenatal patients during the time of the study. The management was as per the institutional protocol conforming to the latest guidelines at the time. Patients underwent radiological evaluation with X-ray, ultrasonography, or CT chest whenever required. The use of steroids and the dosage were guided by the latest guidelines (Royal College of Obstetrics and Gynecology guidelines). The choice of antibiotics was based on local drug sensitivity trends. The ventilatory support if needed was given as per the clinical condition and weaned off as per recovery. All patients on steroids were monitored for blood glucose. and insulin/oral hypoglycemic was added appropriately.

All neonates born to COVID-positive mothers were tested for SARS-CoV-2 in nasopharyngeal and throat samples at the time of birth as per the institutional policy. Maternal and neonatal characteristics including Apgar score, birth weight, neonatal ICU (NICU) admission, respiratory support, neonatal morbidities, and mortality were noted for neonates with early-onset SARS-CoV-2 infection. The presence of SARS-CoV-2 in different maternal samples, i.e., amniotic fluid, cord blood, placental swab, and vaginal and cervical swab collected at the time of birth, was also noted to determine the possibility of mother-to-child transmission.

Statistical analysis

The data analysis was conducted using Statistical Package for Social Sciences (SPSS) Version 21.0 (IBM Corp., Armonk, NY). The continuous data were expressed as mean±SD or median (range), and the categorical data were expressed as a percentage. The continuous data were compared by applying Student’s t-test. The categorical data were compared using Fisher’s exact test. A p-value of <0.05 was considered significant.

## Results

A total of 165 (60, 68, and 37 in the first, second, and third waves, respectively) COVID-positive pregnant women were admitted to the study center. No severe COVID disease during pregnancy was noted in the first and third waves. Likewise, no neonate was found to have early-onset SARS-CoV-2 infection during the first and third waves.

During the second wave (March to June 2021), 15 pregnant women were found to have severe COVID disease and 11 neonates had early-onset SARS-CoV-2 infection.

Pregnant women with severe COVID disease

Among the 68 women in the second wave of infection, maternal and fetal characteristics of 15 pregnant women with severe COVID disease are described in Table 1. The mean age was 29.13±4.01 years, and the mean gestational age at the time of admission was 25.57±4.69 weeks. The majority (73.33%) were in their second trimester of pregnancy, and the rest were in their third trimester.

**Table 1:** Maternal and fetal characteristics of pregnant women with severe COVID disease Data are expressed as mean±SD, n (%), or median (range)

Characteristics	N=15
Age (years)	29.13±4.01
Gestational age (weeks)	25.57±4.69
Trimester	
First	0
Second	11 (73.33)
Third	4 (26.67)
Symptoms	
Fever	9 (60)
Cough	14 (93.33)
Breathlessness	14 (93.33)
Pneumonia	14 (93.33)
Duration of symptoms	4 (2-10)
Clinical assessment	
Heart rate (beats/min)	
<100	7 (46.67)
>100	8 (53.33)
Mean arterial pressure (mmHg)	83±10.53; 81 (75-107)
Respiratory rate (/min)	
<30	4 (26.67)
>30	11 (73.33)
Pregnancy-associated outcomes	
Gestational diabetes mellitus	2 (13.33)
Intrahepatic cholestasis of pregnancy	1 (6.67)
Thyroid disorders	3 (20)
Pre-eclampsia/eclampsia	2 (13.33)
Anemia	12 (80)
Oligohydramnios	5 (33.33)
Intrauterine fetal death	9 (60)

All of these women were symptomatic; most of them (93.33%) had cough, breathlessness, and pneumonia. On admission, seven (46.67%) had tachycardia with an average mean arterial pressure of 83 mmHg. The majority had tachypnea (73.33%) and needed supplementary oxygen right away at the presentation time if not already on it. At presentation, nine (60%) of these women had intrauterine fetal death (IUFD) and all women with IUFD had a critical disease. There was gestational diabetes mellitus in 13.33%, intrahepatic cholestasis of pregnancy in 6.67%, pre-eclampsia/eclampsia in 13.33%, anemia in 80%, and oligohydramnios in 33.33% women.

Table 2 summarizes the management details of the study. All 15 women needed ICU admission; most required invasive ventilatory support. Upon blood investigations, it was observed that nearly three-fourths of the subjects had high total leucocyte count and alanine transaminase, and 100% of them had raised aspartate transaminase. Almost two-thirds of them had normal procalcitonin and increased D-dimer levels. All the patients in this study received anticoagulation prophylaxis, antibiotics, and steroids as part of their treatment.

**Table 2:** Management details of pregnant women with severe COVID disease Data are expressed as mean±SD or n (%) NIV, non-invasive ventilation; SGOT, serum glutamate oxaloacetic transaminase or aspartate transaminase; SGPT, serum glutamate pyruvate transaminase or alanine transaminase

Characteristics	N=15
ICU admission	15 (100)
Ventilatory support	
Face mask/nasal prongs	2 (13.33)
NIV	2 (13.33)
Invasive ventilation	11 (73.34)
Investigations	
Hemoglobin (gm/dL)	10.27±1.36
Total Leucocyte count (/cumm)	13,186.91±4,869.56
<4,000	0
4,000-11,000	4 (26.67)
>11,000	11 (73.33)
Platelet (/cumm)	
<1.5 lakh	3 (20)
>1.5 lakh	12 (80)
Serum creatinine (mg/dL)	0.81±0.24
SGOT (U/L)	186.04±160.35
<40	0
>40	15 (100)
SGPT (U/L)	116.23±104.31
<40	4 (26.67)
>40	11 (73.33)
Ferritin (ug/dL)	347.70±300.62
Procalcitonin (ng/mL)	
<0.5	7/11 (0.64)
>0.5	4/11 (0.36)
D-dimer (ug/mL)	
<0.5	0
0.5-2	5 (33.33)
>2	10 (66.67)
Treatment	
Steroid	15 (100)
Clexane	15 (100)
Antibiotics	15 (100)
Vasopressor	8 (53.33)

Table 3 shows the maternal and fetal outcomes of pregnant women with severe COVID disease. It was observed that all 11 (73.33%) mothers requiring ventilatory support succumbed to death. Only four women during the study period recovered and had a continuation of pregnancy with live fetuses; the outcome after discharge is unknown. Two out of 11 women had a termination of pregnancy resulting in two live births. Both of these neonates were COVID positive in oropharyngeal and nasopharyngeal samples. One of the neonates died within the study duration due to perinatal asphyxia.

**Table 3:** Maternal and fetal outcomes of pregnant women with severe COVID disease Data are expressed as median (range) or n (%)

Characteristics	N=15
Duration of hospital stay (days)	5 (1-11)
Maternal outcome	
Maternal mortality	11 (73.33)
Continuation of pregnancy	4 (26.67)
Fetal outcome	
Intrauterine death	9 (60)
Live births	2 (13.33)
Neonatal death	1/2 (50)
COVID-positive neonate	2/2 (100)

One of the study subjects, primigravida with severe COVID disease at 32 weeks gestation, had a live intrauterine fetus with no evidence of fetal hypoxia. The patient on presentation had respiratory distress needing supplementary oxygen with a face mask. Upon investigation, she had mild anemia, raised liver enzymes, and features of pneumonia. She underwent a cesarean section on day two of admission and was intubated preoperatively as her respiratory alkalosis worsened on the operating table. Upon birth, the neonate was only 1,500 grams with an Apgar score of 2 at 1 minute and 4 at 5 minutes. The neonate died 12 hours after delivery despite ventilatory support, and the cause of death was determined as perinatal asphyxia. The mother’s cardiorespiratory status worsened post-surgery and she needed double inotropic support at high doses. She had an irreversible cardiopulmonary arrest within 36 hours of surgery.

Neonates with early-onset SARS-CoV-2 infection

Table 4 describes neonates' maternal and neonatal characteristics with early-onset SARS-CoV-2 infection. None of them had any COVID-related symptoms. The average maternal age was 29.18 years (range: 27-34 years). The mean parity was 1.54 (range: 1-3). Two mothers had severe COVID disease. The mode of delivery was lower segment cesarean section (LSCS) in nine women, whereas two women had a normal vaginal delivery. The LSCS was commonly performed for obstetric indications except in two cases with severe maternal COVID pneumonia. Preterm birth was reported in four patients. A history of premature rupture of membranes was present in two patients. The average Apgar scores at 1 and 5 minutes were 6.9 (range: 1-9) and 8.09 (range: 4-9), respectively. The average birth weight was 2,551.81 grams (range: 1,375-3,820 grams). All neonates were initially kept in the NICU. Out of 11, four neonates required treatment in the form of positive-pressure ventilation, chest compressions, high-flow nasal oxygen, and non-invasive and invasive ventilation. Neonatal mortality was documented in two cases. The requirement for neonatal interventions and mortality was attributed to prematurity, sepsis, and perinatal asphyxia, rather than SARS-CoV-2 infection.

**Table 4:** Maternal and neonatal characteristics of newborns with early-onset SARS-CoV-2 infection APH, antepartum hemorrhage; CC, chest compression; EONS, early-onset neonatal sepsis; F, female; GA, gestational age; HFNO, high-flow nasal oxygen; LSCS, lower segment cesarean section; M, male; MSL, meconium stained liquor; NIV, non-invasive ventilation, NPOL, non-progression of labor; NVD, normal vaginal delivery; PPV, positive-pressure ventilation; PROM, premature rupture of membrane; PTB, preterm birth; SGA, small for gestational age; SIMV, synchronized intermittent mandatory ventilation

Sl. no.	Age (years)	Parity	Disease severity (maternal)	GA at the time of birth (weeks)	Mode of delivery	Indication of LSCS	PROM >18 hours	PTB	Apgar at 1 minute	Apgar at 5 minutes	Sex	Birth weight (grams)	Neonatal management	Neonatal outcome	Positive results in maternal samples
1	30	2	Mild	39+3	LSCS	MSL	-	-	8	9	M	3,445	-	Stable	-
2	30	1	Mild	41	NVD	-	-	-	8	9	F	2,965	-	Stable	Amniotic fluid, placental swab
3	27	1	Mild	39+3	LSCS	MSL	-	-	9	8	M	2,720	-	Stable	Vaginal and cervical swab
4	34	1	Mild	40+5	NVD	-	-	-	8	9	F	2,700	-	Stable	Vaginal and placental membrane swabs
5	29	3	Mild	29+2	LSCS	APH; placenta previa	-	Yes	1	6	M	1,375	PPV+CC	Stable	Cervical swab
6	27	3	Mild	32+3	LSCS	Anhydramnios	Yes	Yes	8	9	M	1,620	NIV for 20 hrs followed by SIMV for 10 hrs	Death, sepsis (Klebsiella positive EONS)	Vaginal and cervical swab
7	32	2	Mild	37+3	LSCS	Fetal distress	-	-	8	9	M	3,820	-	Stable	-
8	28	1	Severe	32+5	LSCS	Maternal severe COVID pneumonia	-	Yes	2	4	F	1,590	PPV+CC followed by SIMV for 12 hrs	Death (perinatal asphyxia)	Amniotic fluid
9	27	1	Mild	40+1	LSCS	NPOL	-	-	9	9	M	3,135	-	Stable	-
10	24	1	Mild	39	LSCS	MSL	Yes	-	7	8	M	2,625	HFNO for 7 days	Stable	-
11	33	1	Severe	32+5	LSCS	Maternal severe COVID pneumonia	-	Yes	8	9	F	2,075	-	Stable	-

Additionally, the early-onset SARS-CoV-2 infection in neonates was correlated with SARS-CoV-2 in different maternal samples collected at birth to determine the possibility of mother-to-child transmission. Six mothers had one or more positive results in either amniotic fluid, placental membrane, or vaginal or cervical swab, highlighting the possibility of antepartum or intrapartum transmission. The probability of contracting the infection through the environment was almost nil as neonatal samples were taken at the time of birth. SARS-CoV-2 infection can be explained in another five neonates by fetal contamination with maternal blood at the time of LSCS. Breastfeeding was not allowed in COVID-positive mothers; therefore, we cannot comment on postpartum transmission.

## Discussion

This study analyzed the data of 15 pregnant women with severe COVID disease and found them to have a 100% requirement for ICU admission and supplementary oxygen. All were symptomatic and were mostly in their second trimester (73%) and third trimester (27%). Around 60% of these patients had IUFD at presentation. All of these women were also found to have raised aspartate transaminase, and nearly three-fourths had raised alanine transaminase and total leucocyte count. All women requiring invasive ventilation succumbed to the disease with a recorded maternal mortality rate of around 73%. Four women had a continuation of pregnancy. Two women underwent cesarean section for termination of pregnancy. Both neonates tested positive for COVID, out of which only one neonate survived.

Out of 11 COVID-positive neonates, the evidence of mother-to-child transmission was found in only six mothers and had one or more positive results in either amniotic fluid, placental membrane, or vaginal or cervical swab, highlighting the possibility of antepartum or intrapartum transmission [9]. We did not have data on maternal blood and feces contamination with SARS-CoV-2. Therefore, SARS-CoV-2 infection can be explained by fetal contamination with maternal blood and feces at the delivery time in another five neonates. We could not comment on postpartum transmission as breastfeeding was not allowed in COVID-positive mothers.

The concentration of severe COVID disease at later gestations was also observed in a prospective cohort study from the UK with an OR of 3.39 for the second trimester [10]. Unlike the present study, Vousden et al. documented a higher proportion from the third trimester. Similar to the present study, they reported that women with severe diseases mainly required respiratory support and ICU admission. All the maternal deaths were in women having severe diseases. But, there was a higher incidence of pneumonia, intensive care, invasive ventilation, and stillbirths in women in the second trimester, which probably points toward more severity in the second trimester [10].

Raised alanine and aspartate transaminase was a significant finding in the present study. A similar result was noted in a retrospective study from Turkey that included 110 women [11]. They concluded the predictor value of LDH levels to be higher for severe infection. Unfortunately, the data on LDH levels were not available for analysis in the present study. The area under the curve (AUC) for AST and ALT in the Turkish study was 0.840 and 0.771, respectively, but not as high as LDH. Thus, high levels of AST, ALT, or both could strongly predict severe disease. The mean value of AST and ALT observed in the present study was much higher compared to the Turkish research (186.04±160.35, 116.23±104.31, and 50.5, 20, respectively). Different laboratory assays could explain this.

The observed maternal mortality rate of 73.33% is much higher than other studies [12,13]. This could be explained by the fact that the study center deals with high-risk and complicated cases as it is a tertiary referral center close to hilly areas of North India with difficult transportation facilities.

Another study from the same institution comparing the disease parameters in pregnant and non-pregnant cohorts reported that pregnancy per se did not add to morbidity and mortality [3]. Instead, the majority of patients in the pregnant cohort had milder and asymptomatic diseases. This finding was mostly owed to frequent tests and routine admissions. Of the 42 women in the obstetric cohort, 11 (26.19%) had severe disease. An essential finding in the present study was the concentration of severe disease in the second trimester. Even within the pregnancy cohort, when severe and non-severe cases were compared, earlier gestational age in severe cases was a significant finding. Also, all instances of IUFD belonged to the severe disease group [3].

The finding of severe or critical illness in the second trimester raises an important concern for pregnancy with COVID disease in regard to risks of continuation of pregnancy when infected in an early trimester, the timing of termination of pregnancy, mode of termination of pregnancy, or even risk of exacerbation of disease after the termination of pregnancy. The diluted effect of lesser mortality in pregnancy (mostly non-severe illness) compared to non-pregnant women, as observed in many studies [3,12,14], should not be construed as an assurance to the public concerning the effect of COVID disease in pregnancy.

There is a possibility that pregnant women with severe diseases behave much differently than the ones with non-severe diseases. The rate of progression is probably much faster and deadlier [15,16]. Another observation in the present study was the sudden deterioration of general condition soon after the termination of pregnancy, suggesting the possibility of exacerbation of disease postnatally. Similar reports of such incidents have been noted in the literature [17]. The need for termination or the timing of pregnancy termination either to improve the mother’s cardiorespiratory status or to salvage the fetus is a burning question for any obstetrician with insufficient literature to support or negate either [18]. Also, what predicts the severity of this disease is an important research topic of demand. Thus, the pathophysiology of the severe disease in the second trimester needs further evaluation, and more focused studies are in demand to answer the above questions raised during this study.

Strengths and limitations

Detailed evaluation of two aspects of COVID disease, which are rarely studied, including pregnant women with severe COVID disease and neonates with early-onset SARS-CoV-2 infection, is the study's major strength, adding valuable information to the available literature. The detailed protocol will come in handy for the future waves of this deadly virus or its mutations.

There are certain limitations, including the nature of the study, i.e., non-randomized and lack of control group for comparison, and small sample size. As a tertiary care center, most patients were referred from the surrounding centers. Management of these patients was primarily done elsewhere; hence, the quality of care is not uniform across all patients. The data were only limited up until the discharge. Thus the outcome of all the recovered mothers was not available for analysis. At the time of the study, vaccination was not initiated for pregnant women in the country. Thus, the effect or benefit of vaccination cannot be commented upon. The study was only from a single center, and the sample size was too less to generalize the findings across the population. This calls for more focused and comparative studies to make any conclusions.

## Conclusions

Severe COVID disease during pregnancy is present primarily in the second trimester and is associated with a high incidence of intrauterine fetal demise and maternal mortality. Elevated AST, ALT, or both could strongly predict severe disease. Early-onset neonatal SARS-CoV-2 infection is primarily asymptomatic and has a good prognosis. The poor neonatal outcomes can be attributed to prematurity, sepsis, and perinatal asphyxia, rather than SARS-CoV-2 infection. Additionally, mother-to-child transmission of SARS-CoV-2 is possible in the antepartum and intrapartum periods.
